# MicroRNAs are appropriate in mitochondrial related hearing loss? Answer to the skepticism

**DOI:** 10.1186/s13023-018-0865-8

**Published:** 2018-07-18

**Authors:** Arianna Di Stadio, Corrado Angelini

**Affiliations:** San Camillo Hospital IRCCS, Venice, Italy

## Abstract

**Aim:**

We aim to clarify some points that have been criticized about our previous paper “Hearing Impairment in MELAS: new prospective in clinical use of microRNA, a systematic review”.

**Material and method:**

We answered to the criticism of Dr. Finsterer point by point, by citing all literature in support of our previous paper.

**Conclusion:**

The point by point answering allows us to clarify doubts and to support the proposal that we exposed in our review, the possibility to use microRNA for detecting the hearing damage in patients affected from mitochondrial disease.

## Dear Dr. Finsterer,

It is challenging to answer point by point to your remarks in our article [1].

We clearly explained in our paper that we were investigating the use of microRNA (miRs). Our hypothesis is that microRNAs might be an interesting tool to study mitochondrial disorders and its multisystemic manifestations in Mitochondrial encephalomyopathy, lactic acidosis, and stroke-like episodes (MELAS) and we believe that researcher is fully aware of limitations mostly related to the use of preexisting data collected [2].

We stated that additional clinical and experimental studies are necessary. Ideally, a clinical study should link the results of specific audiological and electrophysiological impairments with MELAS and, to the concentration of specific microRNAs. The necessity of prospective studies is clear due to the unspecificity of microRNAs [3], as you correctly underlined.

Our suggested microRNAs are the ones that have been identified in the human temporal bones of patients affected from MELAS [4]; thanks to the high quality of these studies the reported results are consistent and valid [5, 6].

We focused our attention on the variation of concentration of microRNAs only and not, on their multiple functions since this would need more experimental studies.

As far as the influence of aging, we reported that the temporal bones observed came from young subjects (“patients were under 30 years old”) and, in our opinion this is the strength of this research, in fact the “age” may be considered as a confounder if we consider the hearing impairment, that is a common finding in older age. Furthermore ROS that increase with age could be a common pathogenetic link to mitochondrial disorders, since mitochondrial deletions increase with age.

We pointed out the heterogeneity of the human temporal bone and, we underlined different severities of hearing loss observed in MELAS patients are due to the stochastic segregation of mitochondria by determining changes of different entity and that involves different inner ear structures – spiral ganglion, vascular stria [5]. This is well documented by the fact that MELAS patient might have not only lactic acidosis but neurosensory hearing loss or the full MELAS syndrome.

I disagree with you, when you spoke about conductive form of hearing loss (HL) in patients with MELAS, your speculative statement is not correlated with that observed in human clinical observations, patients with MELAS are affected by a sensorineural form of hearing loss even if they present different severity of hearing impairment [1].

The central auditory pathways have been investigated from several authors in patient affected by MELAS [7, 8] by Auditory Brain Response (ABR) for evaluating the damage posterior to the cochlea and their results showed several discordant data [2], so we speculate to use miR-9/9*, that seems to be sensible to identify brain damage and that was validated by experimental data [9].

We included different type of studies in human temporal bones and clinical observations, that we analyzed for explaining that the biomarkers commonly observed in patients affected from MELAS (i.e. creatine kinase (CK), lactic acid) might add to the reported Magnetic Resonance Imaging (MRI) results in the temporal bone analysis.

We stated that hearing loss not only affects patients with MELAS, but also other mitochondrial disorders.

We think that microRNAs could be a valid biomarker for investigating the damage in hearing pathways, currently the method needs to be tested in prospective human clinical studies as first and, then if its accuracy will be confirmed microRNAs might be used in the screening of patients with sensory neural hearing loss.

## Abbreviations

ABR: Auditory Brain Response
CK: Creatine Kinase
HL: Hearing Loss
MELAS: Mitochondrial encephalomyopathy, lactic acidosis, and stroke-like episodes
MiR: MicroRNA
MRI: Magnetic Resonance Imaging

## References

[1] Di Stadio A, Pegoraro V, Giaretta L, Dipietro L, Marozzo R, Angelini C (2018). Hearing impairment in MELAS: new prospective in clinical use of microRNA, a systematic review. Orphanet J Rare Dis.

[2] Egger M, Altman DG, Smith GD. Systematic Reviews in Health Care: Meta-Analysis in Context. 2nd ed. p. 410–8.

[3] Loganantharaj R, Randall TA (2017). The limitations of existing approaches in improving MicroRNA target prediction accuracy. Methods Mol Biol.

[4] Xue T, Wei L, Zha DJ, Qiu JH, Chen FQ, Qiao L, Qiu Y (2016). miR-29b overexpression induces cochlear hair cell apoptosis through the regulation of SIRT1/PGC-1αsignaling: implications for age-related hearing loss. Int J Mol Med.

[5] K T, Merchant SN, Miyazawa T, Yamaguchi T, McKenna MJ, Kouda H, Iino Y, Someya T, Tamagawa Y, Takiyama Y, Nakano I, Saito K, Boyer P, Kitamura K (2003). Temporal bone histopathological and quantitative analysis of mitochondrial DNA in MELAS. Laryngoscope.

[6] Koda H, Kimura Y, Ishige I, Eishi Y, Iino Y, Kitamura K (2010). Quantitative cellular level analysis of mitochondrial DNA 3243A > G mutations in individual tissues from the archival temporal bones of a MELAS patient. Acta Otolaryngol.

[7] Wilichowski E (2001). Progressive sensorineural hearing loss in children with mitochondrial encephalomyopathies. Laryngoscope.

[8] Santarelli RM, Cama E, Scimeni P, La Morgia C, Caporali L, Valentino ML, Liguori R, Carelli V. Reply: both mitochondrial DNA and mitonuclear genemutations can cause hearing loss through cochlear dysfunction. Brain 2016;140:1–5.el. 10.1093/brain/aww052 27016406

[9] Meseguer S, Martínez-Zamora A, García-Arumí E, Andreu AL, Armengod ME (2015). The ROS-sensitive microRNA-9/9* controls the expression of mitochondrial tRNA-modifying enzymes and is involved in the molecular mechanism of MELAS syndrome. Hum Mol Genet.

